# Supplementary material for: Impact on the Transcriptome of Proton Beam Irradiation Targeted at Healthy Cardiac Tissue of Mice
Source: Cancers (Basel). 2024 Apr 11;16(8):1471. doi: 10.3390/cancers16081471 (PMC11048382; doi:10.3390/cancers16081471)
Supplement: Supplementary file 1 [file cancers-16-01471-s001.zip › cancers-2908311-supplementary.pdf]

SUPPLEMENTARY RESULTS OF

Impact on the transcriptome of proton beam irradiation targeted to healthy cardiac tissues of mice.

PRE-PROCESSING. The whole-genome gene expression profiling has been performed and the preliminary results can be summarized as follows: a global analysis of expression profiles in the 23 samples using boxplots does not reveal the presence of samples with macroscopic differences with respect to the others, so the dataset is substantially homogeneous in terms of expression values for each sample. All samples are performed in triplicate (i.e. 3 samples at 72 hours with a dose of 2 Gy, etc.) except for the 10-day control group (2 samples).

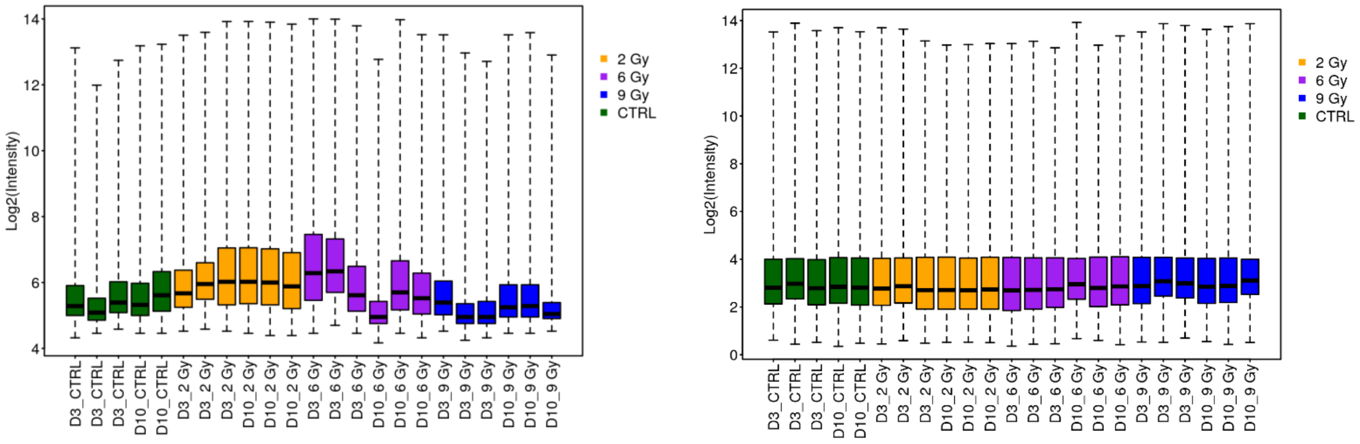

Figure S1. Box plots of expression signal intensity not normalized A) and normalized B). A global analysis of expression profiles in the 23 samples using boxplot does not reveal the presence of outliers or samples with macroscopic differences. Different doses are represented with different colors and each combination of dose-timepoint is represented with its biological and technical replicates.

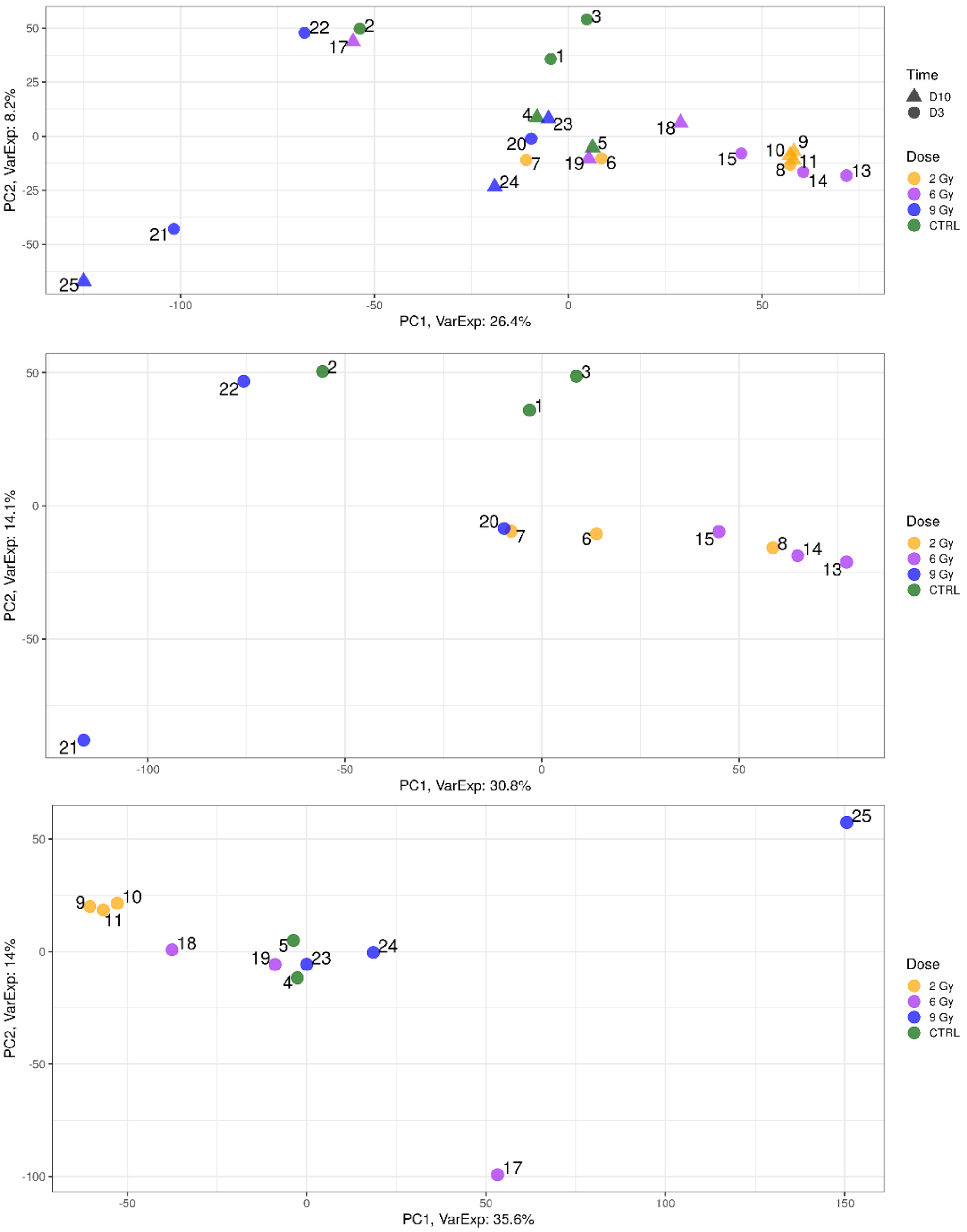

Figure S2. Representation of the samples in the latent space defined by the first two Principal Components (PC1 and PC2) obtained from the Principal Component Analysis (PCA). The percentage of variance explained by each component is reported in the axis label. Samples are colored according to the dose. (Top) Samples from both tie points (T3 and T10) are included. Different time points are represented with different point shapes. (Middle) Only samples from T3 are included. (Bottom) Only samples from T10 are included.

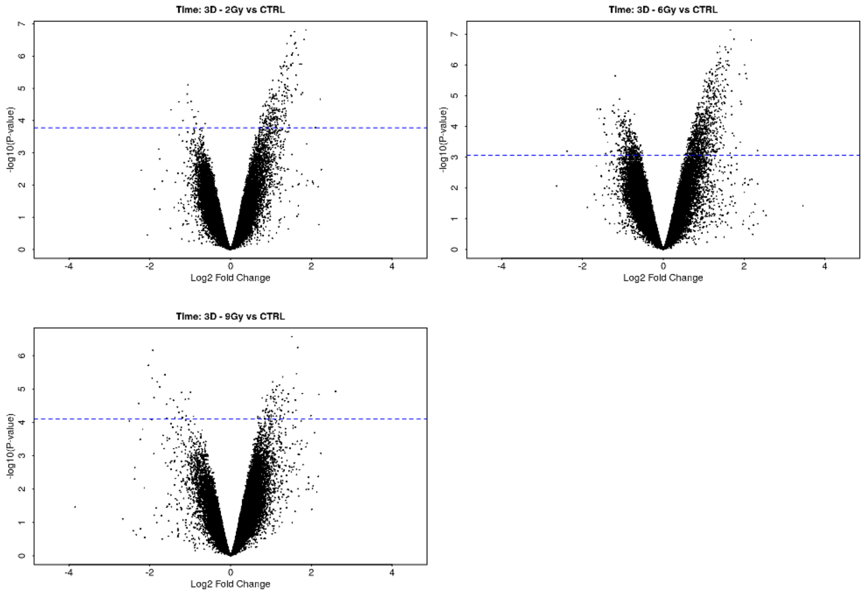

Time: 3D - 2Gy vs CTRL
Time: 3D - 6Gy vs CTRL
Time: 3D - 9Gy vs CTRL
-log10(P-value)
Log2 Fold Change

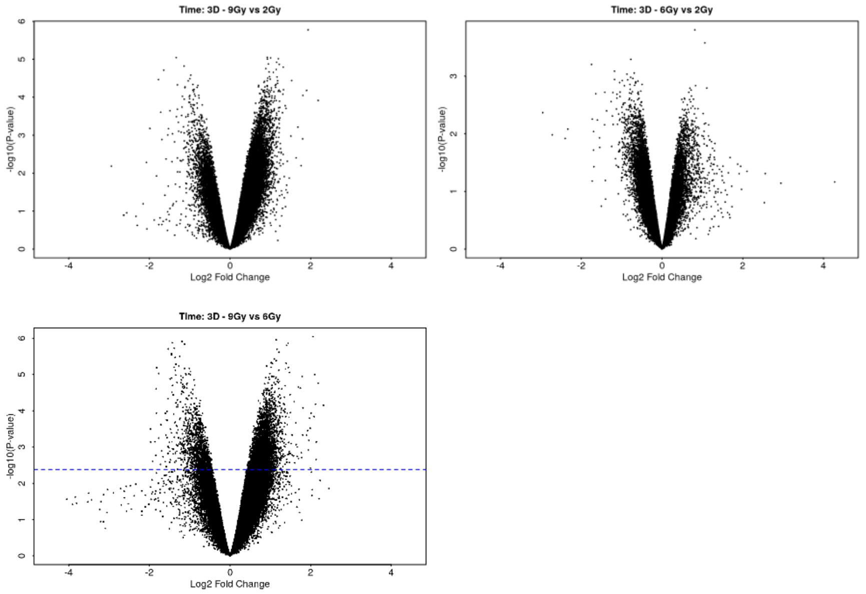

Time: 3D - 9Gy vs 2Gy
Time: 3D - 6Gy vs 2Gy
Time: 3D - 9Gy vs 6Gy
-log10(P-value)
Log2 Fold Change

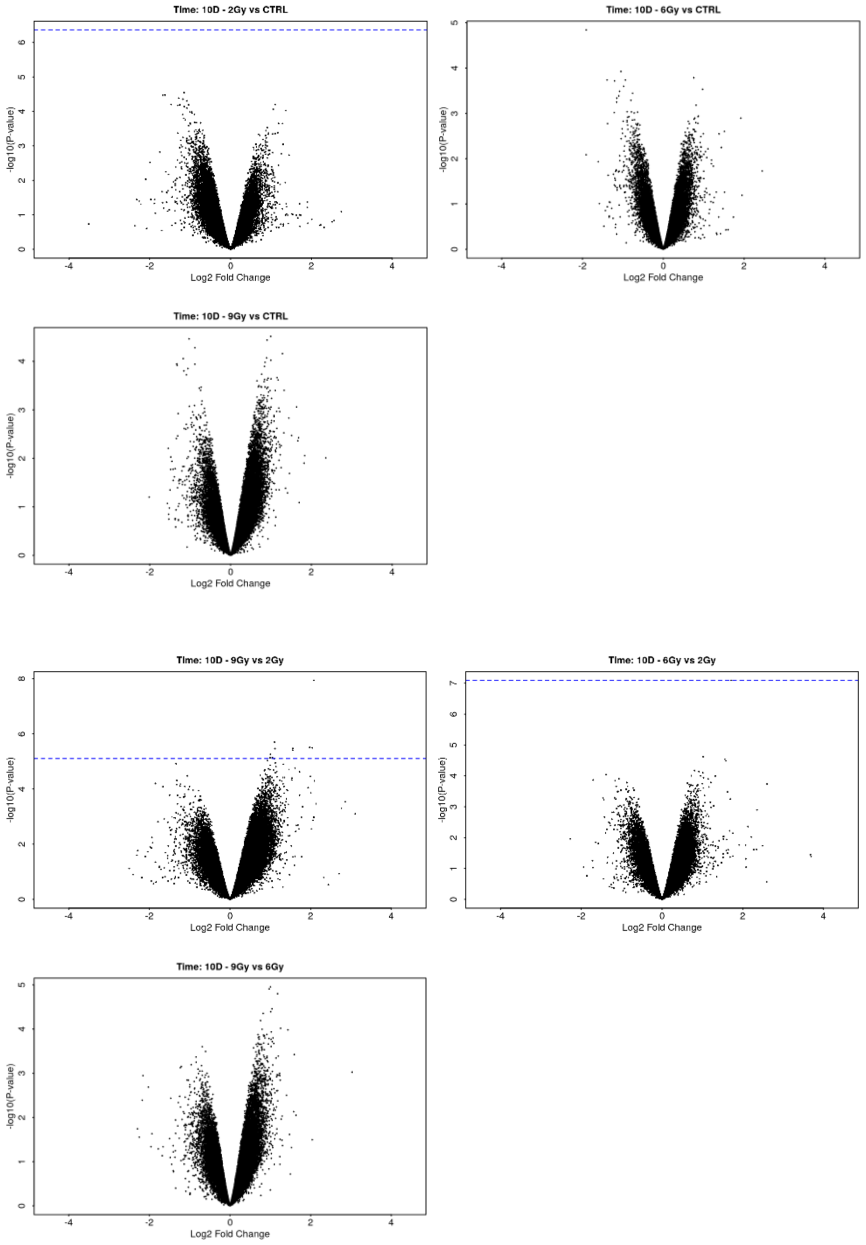

Figure S3. Volcano plots show the statistical significance (- $\log_{10}$ (P-value)) versus the magnitude of LFC for each comparison. A dashed blue horizontal line is drawn to highlight the significance threshold corresponding to an adjusted P-value of 0.05.

Table S1. List of DEGs coding for proteins of interest in pathways relevant for either cell cycle regulation or transcription regulation, cellular metabolism and vesicle trafficking, or in the cardiac tissue. In the table are reported the gene symbol, the full gene name and a summary of the molecular function played by the gene.

**Gene symbol**	**Gene Name**	**Function**
Cdkn1a	Cyclin-dependent kinase inhibitor 1A	Highly conserved cell division cycle protein that functions as a serine/threonine protein kinase, playing a regulatory role in DNA replication and damage repair by promoting or inhibiting the progression at G1 and S phase entry preparation.
Trp53inp1	Tumor protein p53-inducible nuclear protein 1	Antiproliferative and proapoptotic protein involved in cell stress response which acts as a dual regulator of transcription and autophagy. Acts as a positive regulator of autophagy.
Hsp90aa1	Heat shock protein 90, alpha, class A member 1	Molecular chaperone, involved in the structural maintenance and proper regulation of Cdkn1a and others specific target proteins involved in cell cycle control and signal transduction.
Eda2r	Ectodysplasin A2 receptor	Tumor necrosis factor receptor (TNFR) that mediates the activation of the NF-kappa-B and JNK pathways, therefore acting as signal transduction in apoptosis and inflammation responses.
Bhlhe40	Basic helix-loop-helix family, member e40	Transcriptional repressor involved in the regulation of the circadian rhythm by negatively regulating the activity of the clock genes and clock-controlled genes. Drives the circadian rhythm of blood pressure through transcriptional repression of *ATP1B1* in the cardiovascular system (PubMed:30012868)
Uprt	Uracil Phosphoribosyltransferase Homolog	Catalyzes the conversion of uracil and PRPP to uridine monophosphate (UMP). This reaction is an important part of nucleotide metabolism, specifically the pyrimidine salvage pathway. The protein is a potential target for rational design of drugs to treat parasitic infections and cancer.
Lamp2	Lysosome-associated membrane glycoprotein 2	Involved in in chaperone-mediated autophagy, a process that mediates lysosomal degradation of proteins in response to various stresses and physiological turnover.
Ogn	Osteoglycin	In synergy with others non-structural matrix-cellular proteins (MCPs) including TSP-2 and SPARC modulate the process of heart failure (HF) and cardiac inflammation.
Vamp7	Vesicle Associated Membrane Protein 7	Encodes a transmembrane protein that is a member of the soluble N-ethylmaleimide-sensitive factor attachment protein receptor (SNARE) family. The protein localizes in late endosomes and lysosomes and is involved in the fusion of transport vesicles to their target membranes,
Hspg2	Heparan sulfate proteoglycan 2 (aka Perlecan, PLC)	Large multidomain proteoglycan that binds to and cross-links many extracellular matrix (ECM) components and cell-surface molecules. It is synthesized by both vascular endothelial and smooth muscle cells and deposited in the extracellular matrix
Flnc	Filamin C	Muscle-specific filamin that plays a central role in muscle cells, probably by functioning as a large actin-cross- linking protein. These filamin proteins crosslink actin filaments into orthogonal networks in cortical cytoplasm and participate in the anchoring of membrane proteins for the actin cytoskeleton.
Ltbp4	Latent-transforming growth factor beta-binding protein 4	Key regulator of transforming growth factor beta that controls TGF-beta activation by maintaining it in a latent state during storage in extracellular space.
